# Centralisation of acute obstetric care in the Netherlands: a qualitative study to explore the experiences of stakeholders with adaptations in organisation of care

**DOI:** 10.1186/s12913-021-07269-4

**Published:** 2021-11-13

**Authors:** Lauri M.M. van den Berg, Bernardus Benjamin Maria Gordon, Sophia M. Kleefstra, Lucie Martijn, Jeroen van Dillen, Corine J. Verhoeven, Ank de Jonge

**Affiliations:** 1 Department of Midwifery Science, AVAG/Amsterdam Public Health, Amsterdam University Medical Centre, Vrije Universiteit Amsterdam, Van der Boechorststraat 7, 1081 BT Amsterdam, the Netherlands; 2 Department of Obstetrics and Gynaecology, Radboud University Medical Centre, Geert Grooteplein Zuid 10, 6523 GA Nijmegen, the Netherlands; 3 Dutch Health and Youth Care Inspectorate, Stadsplateau 1, 3521 AZ Utrecht, the Netherlands; 4 Maxima Medical Centre, Department of Obstetrics and Gynecology, De Run 4600, Veldhoven, Netherlands; 5 Division of Midwifery, School of Health Sciences, University of Nottingham, Floor 12, Tower Building, University Park, NG7 2RD Nottingham, UK

**Keywords:** Maternity care, Acute obstetric care, Centralisation, Organisation of maternity care, Stakeholder involvement, Change management, Thematic analysis

## Abstract

**Background:**

In the past decade, acute obstetric care (AOC) has become centralised in many high-income countries. In this qualitative study, we explored how stakeholders in maternity care perceived and experienced adaptations in the organisation of maternity care in areas in the Netherlands where AOC was centralised.

**Methods:**

A heterogenic group of fifteen maternity care stakeholders, including patients, were purposively selected for semi-structured interviews. An inductive thematic analysis was used.

**Results:**

Three main themes were identified: (1) lack of involvement. (2) the process of making adaptations in the organisation of maternity care. (3) maintaining quality of care. Stakeholders in this study were highly motivated to maintain a high quality of maternity care and therefore made adaptations at several organisational levels. However, they felt a lack of involvement during the planning of centralisation of AOC and highlighted the importance of a collaborative process when making adaptations after centralisation of AOC.

**Conclusions:**

Regions with AOC centralisation plans should invest time and money in change management, encourage early involvement of all maternity care stakeholders and acknowledge centralisation of AOC as a professional life event with associated emotions, including a feeling of unsafety.

**Supplementary Information:**

The online version contains supplementary material available at 10.1186/s12913-021-07269-4.

## Background

In the past decade, acute obstetric care (AOC) has become centralised in many high-income countries, such as the Netherlands. This has occurred for several reasons [1–3]. First, hospitals have merged to increase their expertise, funding and bargaining power. Second, some hospitals in the Netherlands have gone bankrupt. Finally, Dutch maternity care had several medical workforce problems, such as a lack of paediatricians, which led to closure of obstetric care units. In 2014, 84 hospitals in the Netherlands offered 24/7 acute obstetric care [4]; in 2020, this number had decreased to 74 [5]. Nevertheless, in 2020 99.8 % of Dutch women lived within 45 min travel time to the nearest hospital [5].

Various studies have assessed the benefits and risks of centralisation. Benefits include the full-time availability of medical professionals (such as obstetricians, paediatricians, anaesthetists) and therefore easy access to interventions during birth and rapid identification and management of mothers and neonates with unexpected morbidities [2, 6]. Several studies have found better perinatal outcomes in communities with large obstetric care facilities, especially for very premature neonates [2, 7, 8]. However, studies done in France and Canada concluded that women living far from a hospital (≥ 45 km or ≥30 min) did not have an increased risk of perinatal mortality [3, 9, 10]. In Finland, perinatal outcomes between women living in areas with higher-level perinatal units and areas with small perinatal units were similar [1].

The risks of centralisation of AOC include a longer travel time, less continuity of care and possibly an increase in interventions and costs [1, 3, 8, 11–17]. A Dutch study showed a significant association between a travel time from home to the hospital of 20 min or more and an increased risk of perinatal mortality, in particular for women in obstetrician-led care at the onset of labour [11]. A Canadian study found more maternal mortality and morbidity in women who had to travel more than 200 km to a hospital [8]. Moreover, a travel time to the hospital of more than one hour will increase the risk of unplanned out-of-hospital births, which may have a relation with adverse perinatal outcomes [1, 3, 12]. Another risk of centralisation might be that in high-risk-oriented hospitals, low risk women might have an increased risk of obstetric interventions; however, studies are inconclusive about this [13–15]. Furthermore, financial costs tend to increase when perinatal units become larger [16, 17]. Finally, the centralisation of AOC might reduce choice in place of birth, a choice considered valuable by women [18–20]. Considering all the evidence, there is no clear-cut answer to whether centralisation of AOC is supported or not by the scientific literature, especially since there are differences in the extent of centralisation [21].

The maternity care system in Netherlands differs from that offered in most other high-income countries. This may limit the generalisability of most published international research about the centralisation of AOC. In the Netherlands, women at low risk of complications start their antenatal care with a primary care midwife (87 % in 2019) [22]. When risk factors or complications arise, women are referred to secondary care. In 2019 42 % of women were referred to secondary care during pregnancy [22]. This secondary care is provided by clinical midwives and (trainee) obstetricians. Close collaboration between primary and secondary care is organised in Maternity Care Collaborations (MCCs) around each hospital [23].

Maternity care providers have made adaptations in the organisation of care after centralisation of AOC, such as reorganising MCCs and implementing better back-up arrangements in primary care, for instance by another midwife or an ambulance nearby, because of longer travel times [24, 25]. As far as we are aware, there is no scientific literature about the way in which maternity care stakeholders, including patients, deal with the adaptations in the organisation of maternity care after the centralisation of AOC and how these adaptations affect them. In order to contribute to appropriate supervision on maternity care, the Dutch Health and Youth Care Inspectorate (HYCI) commissioned this study. We aimed to gain insight into the experiences of maternity care stakeholders, since this knowledge might be important for the success of future centralisation of AOC. Therefore, we performed this qualitative study with the following research question: what are the perceptions and experiences of stakeholders regarding the adaptations in the organisation of maternity care after centralisation of AOC?

## Methods

### Study design

Given the unexplored nature of the topic, we conducted an exploratory semi-structured interview study, using a thematic analysis [26]. We selected this analysis method because it offers flexibility and theoretical freedom to explore experiences, meanings and the reality of participants [26]. As this is a qualitative study, prior registration of the study protocol was not mandatory. The protocol was developed with the research team prior to the start of the data collection (Supplementary File 1).

### Setting and participants

Three regions in the Netherlands which recently faced centralisation of AOC (< 3 years ago) were selected. Two regions had to close AOC units due to medical workforce problems: Treant hospital locations in Stadskanaal and Hoogeveen and St. Antonius hospital Nieuwegein; the third region after the bankruptcy of the hospital MC IJsselmeerziekenhuizen Lelystad. Two regions are rural with small towns and less than 1000 births per year per hospital (Treant and MC IJsselmeerziekenhuizen) and one region is an urbanised region with more than 1000 births per year per hospital (Nieuwegein).

A heterogenic group of fifteen stakeholders, including patients (*n*=3) and care providers from the selected regions (*n*=7), were purposively selected for semi-structured interviews (Table 1). The purpose of the sampling was to reach a good representation of the stakeholders involved in maternity care. They were involved in maternity care nationally and/or in the three selected regions. All interviewees participated in a personal capacity. Two interviewees had a double role. To secure anonymity, these double roles are not further described, since the combination of these specific roles in a small country like the Netherlands is unique to a specific person.

**Table 1:** Participants interviews

Stakeholders	n
Health and Youth Care Inspectorate (HYCI)	3
Dutch College of Perinatal Care (CPZ)	1
Health care insurance company	1
Primary care midwife	4
Clinical midwife	2
Obstetrician	1
Maternity care assistants organisation	1
Netherlands Patients Federation	1
Patients	3

Two participants had a double role

 The participants were approached by e-mail. The interviews were held from March to May 2020 (LB). One interview was conducted at the workplace of the participant, and the other fourteen were held by telephone due to Dutch government regulations during the COVID-19 pandemic. The interviewer is a registered midwife and never worked in the selected regions. This was the first study on centralisation of AOC she conducted. There was no previous relationship between the interviewer and the interviewees.

### Data collection

The data collection method was semi-structured interviews, conducted in Dutch. Before the interviews were conducted, an interview guide was made based on an extensive literature search of scientific literature and policy papers (Supplementary File 2). Moreover, the theoretical models of managing complex change, Lewin’s change model curve and the Kübler-Ross change curve were used to develop the interview guide [27–29]. These models were used to understand why and how people and organisations change. Before the data collection of this study started, the interview guide was pilot tested in two pilot interviews, one with a primary care midwife and one with a patient. These interviews were not included in this study, but were used to make the interview guide more comprehensible. The interviews were audio-recorded, and the interviewer kept a diary during and after the interviews to gain insight into her own observations and thinking process.

### Data analysis

The data analysis was an iterative process, with alternating phases of interviewing and analysing. The interviews were transcribed and anonymised (LB) in order to conduct a thematic analysis. The transcripts were analysed with an inductive thematic analysis process according to Braun and Clarke [26]. The software package ATLAS.ti v.8 was used for this. Two researchers (LB, SK) closely read the first interview and formulated initial codes independently. After this process, they compared the codes and reached consensus through discussion. The initial codes were used as a basis to code the other interviews and to recognise patterns [26]. After this, the different initial codes were sorted into overarching themes [26]. Research team meetings were organised to review, refine and define the overarching themes [26]. A third researcher (BG) closely read all fifteen interviews and made summaries. These summaries were used to verify the open codes and formulated themes. Disagreements were solved through extensive discussion. The writing process was guided by the Consolidated Criteria for Reporting Qualitative Research (COREQ) [30]. Since the interviews were held in Dutch, the quotes were translated in collaboration with the entire research team.

## Results

Data saturation was reached after fourteen interviews. Three main themes were identified from the interviews. The first theme was lack of involvement of care providers and patients. The second theme was the process of making adaptations in the organisation of maternity care. The third theme was maintaining quality of care, with the subthemes safety, vision on maternity care and innovative adaptations.

### Lack of involvement of care providers and patients

All of the care providers and patients in this study indicated that there was a lack of involvement during the process of centralisation of AOC, and that early involvement of care providers and patients is vital in the process of centralisation of AOC. For some stakeholders, it was important to have insight into the decision-making process and to participate before the definitive decision to close an AOC unit was taken. Other stakeholders defined involvement as being welcomed in a new setting.

‘But as a Maternity care assistant organisation we just weren’t asked [for input in an early phase] ... Actually, we didn’t have any say’. (Employee Maternity Care Assistant Organisation)

‘They [obstetricians] showed the intention to, well, welcome us. Certainly in the beginning, when the hospital went bankrupt, the obstetricians actively approached us. They told us they would like to hear it if they could help and things like that. So they did show a gesture of goodwill’. (Primary care midwife)

In addition, interviewees stated that existing constructive collaboration between care providers is helpful for the involvement of care providers. Existing constructive collaboration could be a well-organised MCC, but also informal contacts such as ‘knowing each other’.

An inhibiting factor for involvement is acute centralisation of AOC, which led to less time and willingness for involvement of care providers and patients. Furthermore, most of the interviewees stated that maternity care providers were not educated to manage such a complex change after centralisation of AOC. Several participants mentioned the importance of people with experience in managing complex change, and some clinics and midwifery practices appointed care providers to focus on organisational changes, which was considered to be helpful.

‘We ask too much of the maternity care providers in my opinion. You are educated as a midwife and in a way you need to be a director of a MCC as well’. (Employee College of Perinatal Care)

Patient involvement is important according to the interviewees and succeeds or fails with the provision of good information. Most of the interviewees, including the patients, felt that the care providers in an MCC should collaborate more in sharing information with patients in order to prevent conflicting information. Moreover, it was stated several times that information must be adjusted to patients and not be too difficult to understand.

‘But, maybe, the way they [the municipality] offer information, there is quite a mismatch between what they probably do and how it comes through to the target audience [pregnant women]’. (Employee Netherlands Patients Federation)

### The process of making adaptations in the organisation of maternity care

Several participants suggested that the process of making adaptations was a vital part of their experience with these adaptations. They argued that a collaborative experience with the process led to more acceptance of the adaptations and contributed to feeling proud of the achieved adaptations.

**Fig. 1:**
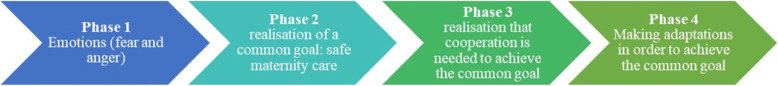
Visual presentation of the process of making adaptations after the centralisation of AOC inspired by the model of managing complex change, Lewin’s change model curve and the Kübler-Ross change curve [27–29]

Figure 1 provides a visual presentation of the process of making adaptations in maternity care after centralisation of AOC as described by the interviewees. Interviewees mentioned that the first response after closure of an AOC is often an emotional response. Closure of an AOC can be a professional life event for care providers. When an AOC unit closed, care providers and patients experienced several emotions, including fear and anger. Care providers noted fear of adverse maternal and neonatal outcomes and a midwife experienced fear of bankruptcy of her own practice as well. Some interviewees were angry, mostly about decisions and adaptations made without their involvement. If the closure of an AOC unit occurred suddenly the emotions were more tense, and it took longer to accept the new situation. This also applied to the patients who were interviewed.

‘And because we were suddenly confronted with the closure, well, it was quite a thing. The world was upside down for a while’. (Primary care midwife)

‘And really a few weeks later we heard that the hospital went bankrupt. Well, for a moment I really didn’t know what to do, I was in quite a panic’. (Patient)

After the initial emotions, the knowledge that centralisation was mostly unavoidable, for example because of bankruptcy or a lack of personnel, led to acceptance (phase 2). The realisation of their common goal to maintain safe maternity care made cooperation between stakeholders possible, which is phase three.

‘Can I do something about it [the closure of the AOC unit]? Well, no, I have to go on, because we just don’t have enough paediatricians. So you just want to make adaptations in the care you deliver’. (Clinical midwife)

‘Eventually, you have to cooperate with each other [all maternity care providers] in a positive way … And that generated a lot of emotions, it definitely wasn’t without struggles.’ (Obstetrician).

In phase four, care providers made adaptations themselves and in cooperation with other care providers, such as in a new MCC, or with other stakeholders (Table 2). This phase can be more difficult when care providers have conflicting financial interests. Interviewees argued that sometimes, ‘the best for the patient’ is not the best for the financial security of the care provider, which can lead to conflict.

**Table 2:** Adaptations after centralisation of AOC mentioned by the interviewees in this study [in alphabetical order]

Adaptations at a national level	Adaptations at a regional level	Adaptations at a local level
Additional allowance for midwifery practices when there is reduces availability or accessibility of secondary care	Better back-up agreements between care providers	Always a room available in the hospital for emergencies in primary care
	Compensation of additional travel costs for patients by the municipality	Better back-up agreements in own organisation
	Harmonising protocols in the region	Discouraging homebirths when travel distance is long
	Increased ambulance capacity	Early call to maternity care assistant organisation for delivery assistance
	Innovative adaptations, such as antenatal CTG in primary care	Hire more staff in a midwifery practice/in the hospital
	Monitoring capacity in the hospitals, for example with an app	Midwife on call in primary care sleeps in her work region
	Redesigning Maternity Care Collaboration	Offer elective inductions for women with a long travel distance to the hospital
		Travel distance as a factor in decision making
		Triage room(s) in the hospital
		Two midwifery teams in a midwifery practice
		Health insurance coverage for outpatient birth

‘What has been the win-win situation mainly was that they buckled down together’. (inspector HYCI)

‘So you know, that is, when the discussion moved to that direction [money], suddenly all the doors closed’. (Obstetrician)

Information about how regions adapted to centralisation of AOC and how people communicated about these adaptations was considered to be valuable for the process of making adaptations in regions that may face centralisation in the future. Participants were unanimous in the view that sharing good practices and information about what worked and what did not could be helpful for other regions.

‘Why isn’t there a clear story about how people dealt with it in that region, how did this end, did it work well or not and does it fit here, yes or no. I missed that from a practical perspective’. (Clinical midwife)

Some participants thought a national organisation, such as the College of Perinatal Care (CPZ), should share good practices, while others were in favour of a regional organisation such as the Regional Counsel Acute Care (ROAZ).

### Maintaining quality of care

Stakeholders in this study were highly motivated to maintain a high quality of maternity care after centralisation of AOC. Safety, vision on maternity care and innovative adaptations were aspects of maintaining quality of care after centralisation of AOC.

#### Safety

Whilst a minority of interviewees mentioned that there were some near misses, all agreed that in general the maternity care remained safe after the centralisation of AOC. All care providers indicated that they make different decisions when they are aware of a longer travel distance to the hospital. Some interviewees proposed that travel distance should be addressed in guidelines as a decision factor.

‘You adjust your policy based on it [travel distance]. So no, I don’t think maternity care has become less safe because of it [the centralisation of AOC]’. (Primary care midwife)

However, at the same time many care providers mentioned feeling unsafe, which for some was a threat to their job satisfaction. This feeling was more prominent amongst care providers working in regions with capacity problems. One care provider mentioned that she always started her shift with a feeling of fear that all the hospitals in her region would be at full capacity. Some care providers felt lonely with this feeling.

‘Where will I end up today, how are things regulated over there? I hope nothing happens when I am out of my region. Is the ambulance coming in time? Well, the feeling of, yeah, not having as much control as we used to have. And that is, that is just a psychological and mental thing we all struggle with’. (Primary care midwife)

Finally, one clinical midwife mentioned that changes in work team after centralisation of AOC could be a risk factor for suboptimal performance of maternity care professionals and advised team building for optimising the collaboration within a newly formed team.

#### Vision on maternity care

Some interviewees felt that quality of care based on their vision on maternity care was compromised after centralisation of AOC. Especially primary care midwives mentioned the importance of continuity of care. Employing more midwives to deal with longer distances reduced continuity of carer in their view or led to the creation of more teams of midwives in order to prevent this from happening.

Both an increase and decrease in the number of inductions were mentioned as undesirable because the decisions were made on non-clinical grounds. More inductions were carried out to prevent unintended out-of-hospital births and fewer to reduce capacity problems due to prolonged admissions.

‘If someone lives next door to the hospital in [city name] you are sometimes somewhat more liberal in the choices you make’. (Obstetrician)

Most of the participants believed that the option of a home birth should remain in regions with a concentration of AOC, possibly guaranteed by more ambulance capacity. However, some stakeholders mentioned that the possibility to have a home birth should not be a goal in itself and suggested that planned home births should no longer be an option in some centralisation regions. The prolonged travel distance to a hospital could make it both more and less likely for patients that were interviewed to plan a home birth.

‘If the midwife visits to check up on me and I was, let’s say, already nine centimetres dilated than I wouldn’t go all the way to [city name hospital], no, I wouldn’t ... Because I can’t recommend you a car ride with contractions’. (Patient)

#### Innovative adaptations

A big change such as the closure of an AOC unit can make it easier to discuss former agreements, especially when a new MCC has to be formed. One way to improve the quality of maternity care is to implement innovative adaptations. Most interviewees were in favour of implementing innovative adaptations, such as antenatal cardiotocography (CTG) in primary care.

‘What we now see is more cooperation regarding performing external cephalic version, regarding the [antenatal] CTG in primary care, which is taking off more quickly in centralisation regions, also to limit travel time for the pregnant woman. And so you also see that larger MCCs get more done in terms of innovation.’ (inspector HYCI).

## Discussion

### Key findings

The aim of this study was to explore the perceptions and experiences of stakeholders regarding the adaptations in the organisation of maternity care after centralisation of AOC. Care providers and patients felt a lack of involvement in the process of centralisation of AOC and indicated that a collaborative process contributed to a positive attitude towards adaptations. While the interviewees stated that in their view maternity care remained safe after the centralisation of AOC, there was a feeling of unsafety amongst them. They argued that this feeling of unsafety could lead to less job satisfaction and feelings of fear and loneliness. Furthermore care providers sometimes felt that adaptations made in the interest of safety sometimes conflicted with their vision on quality of maternity care. Interviewees supported sharing good practices, with the acknowledgement that every region is different and there is no ‘one-size-fits-all’ approach.

### Comparison with previous literature

Most care providers did not feel that they had the skills to lead the process of making adaptations after centralisation of AOC, hence the advice of some interviewees was to invest (money/time) in people to lead the process of making adaptations. Change management is a skill that is necessary to overcome resistance to change and achieve successful and well-accepted long-term changes in an organisation or system [31, 32]. People are more resilient to change when they understand the rationale behind the change [33]. An expected outcome of this study was therefore that care providers and patients indicated that early involvement in the decision-making process was important during centralisation of AOC, a finding supported by the Dutch Council for Public Health and Society [34]. Furthermore, acute closure of an AOC unit has too many disadvantages for involvement and collaboration of care providers and patients according to the interviewees and must be prevented at all times, both for the support of the closure and for patient safety [35].

An interesting finding is the shared agreement that maternity care remained safe after the centralisation of AOC. It is mandatory for care providers in the Netherlands to report serious incidents to the Health and Youth Care Inspectorate [36]. The HYCI has not received any reports on serious incidents related to patient unsafety in the regions that have centralised AOC, which supports the impression that maternity care remained safe after centralisation of AOC [24]. However, the feeling of unsafety experienced by the care providers and patients in this study was clearly evident. This feeling of unsafety could have an impact on the emotional wellbeing of care providers and their risk for burn-out [37, 38]. Care providers with a burnout are at higher risk of contributing to reduced quality of patient care and have a higher risk of developing depression and retiring early, which is a considerable loss given the current medical workforce problems [38]. It is important to acknowledge that the emotional wellbeing of care providers plays a vital role in a healthy maternity care system and that it is therefore urgent to recognize and act on this feeling of unsafety that is experienced by care providers in centralisation regions.

Some interviewees mentioned that centralisation of AOC influenced the decisions during pregnancy and birth that care providers and patients made regarding induction of labour because of increased travel distance to the hospital or restrictions in home birth facilities. This means that besides clinical rationale and initial preferences of the patient, organisational circumstances influence the content of maternity care. Maternity care is already influenced by non-clinical factors. For example, the use of prenatal care is influenced by health care system and personal barriers [39]. Moreover, staffing patterns influence caesarean section rates [40]. Centralisation of AOC may add to this. It is very important to keep in mind patients’ interests when organisational changes are made after centralisation of AOC and be aware that these may reduce ‘high value care’, such as continuity of care [41–43].

### Implications for practice, supervision and research

It is very well possible that AOC will be centralised even more in the future. This study showed that closure of AOC units must be handled with care. The most important preconditions are preventing acute closure, paying attention to the emotions of care providers and patients and involving all maternity care providers, not just the ones working in the closing AOC unit, in decision-making. These preconditions can be used as recommendations by an inspectorate in regions with centralisation plans, especially when there are signals that these preconditions are not met. Further quantitative research on perinatal outcomes in Dutch centralisation regions is recommended, since this could contribute to a more objective discussion about safety after centralisation of care and might lessen the feeling of unsafety of care providers.

**Fig. 2:**
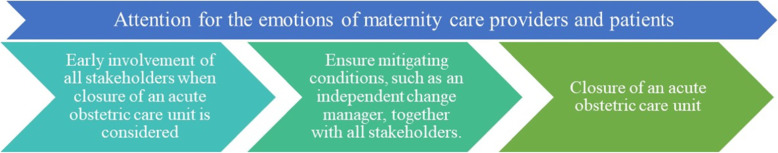
Blueprint for a smooth centralisation of AOC with involvement of stakeholders

We developed a blueprint for a smoother centralisation of AOC (Fig. 2), that can be used in future centralisation regions. After closure of an AOC unit continuous improvement in quality of care in centralisation regions is needed with involvement of all stakeholders.

### Strengths and limitations

Our study has some strengths and limitations. The interviews were held with several stakeholders, including patients, which provided a broad and realistic impression of opinions at the patient, professional and management levels. Furthermore, the interviewer was a primary care midwife, which was an advantage in the interviews with the professional stakeholders, since she had a thorough understanding of the emotions in the field and it was possible to use jargon. In order to avoid focussing merely on primary care, a multidisciplinary research team was involved. Three researchers of the team were involved in the data analysis and discussed codes and themes until consensus was reached, which heightened the credibility of this study as well. A limitation of this study was that the data collection was influenced by the COVID-19 pandemic. Due to government regulations, fourteen of the fifteen interviews were held by telephone. Telephone interviews might decrease the quality and quantity of the data due to the absence of nonverbal or visual cues. Nevertheless, the COVID-19 health crisis was an extremely busy time for care providers, so the telephone interviews were convenient for them and increased their willingness to participate. Another limitation is that some of the results of this study might be more difficult to generalise to countries without a community based maternity care system or to larger countries than the Netherlands, with longer travel times and distances to hospitals.

## Conclusions

This qualitative study has increased our understanding of how maternity care changed after the centralisation of acute obstetric care in certain regions in the Netherlands. Stakeholders in this study were highly motivated to maintain high quality of maternity care and made adaptations at several organisational levels. It should be a priority in regions with AOC centralisation plans to prevent acute closure, to encourage early involvement of all maternity care stakeholders including patients, to invest time and money in change management and to recognise centralisation of AOC as a professional life event with associated emotions, including a feeling of unsafety. Learning of “good practices” in regions that experienced centralisation before might be helpful.

## Supplementary Information

**Additional file 1.**

**Additional file 2.**

## Data Availability

The datasets generated and/or analysed during the current study are not publicly available due to protection of anonymity of the participants but are available from the corresponding author on reasonable request.

## Abbreviations

AOC: Acute obstetric care
CPZ: Dutch College of Perinatal Care
CTG: Antenatal cardiotocography
HYCI: Dutch Health and Youth Care Inspectorate
MCC: Maternity Care Collaboration
ROAZ: Regional Counsel Acute Care

## References

[1] Hemminki E, Heino A, Gissler M (2011). Should births be centralised in higher level hospitals? Experiences from regionalised health care in Finland. BJOG.

[2] Moster D, Lie RT, Markestad T (2001). Neonatal mortality rates in communities with small maternity units compared with those having larger maternity units. BJOG.

[3] Pilkington H, Blondel B, Drewniak N, Zeitlin J (2014). Where does distance matter? Distance to the closest maternity unit and risk of foetal and neonatal mortality in France. Eur J Public Health.

[4] Kommer, GJ, Gijsen, R, Lemmens, LC, Kooistra, M, Deuning, C. Beschikbaarheid, specialisatie en bereikbaarheid van Spoedeisende hulp in Nederland. Utrecht: National Institute for Public Health and the Environment; 2015. https://www.rivm.nl/bibliotheek/rapporten/2015-0077.pdf. Accessed 23 Jul 2021.

[5] National Institute for Public Health and the Environment. Bereikbaarheidsanalyse SEH’s en acute verloskunde. Utrecht: National Institute for Public Health and the Environment; 2020. https://www.rivm.nl/documenten/bereikbaarheidsanalyse-sehs-en-acute-verloskunde-2020. Accessed 23 Jul 2021.

[6] Thellesen L, Sorensen JL, Hedegaard M, Rosthoej S, Colov NP, Andersen KS (2017). Cardiotocography interpretation skills and the association with size of maternity unit, years of obstetric work experience and healthcare professional background: a national cross-sectional study. Acta Obstet Gynecol Scand.

[7] Heller G, Richardson DK, Schnell R, Misselwitz B, Kunzel W, Schmidt S (2002). Are we regionalized enough? Early-neonatal deaths in low-risk births by the size of delivery units in Hesse, Germany 1990-1999. Int J Epidemiol.

[8] Aubrey-Bassler FK, Cullen RM, Simms A, Asghari S, Crane J, Wang PP (2019). Population-based cohort study of hospital delivery volume, geographic accessibility, and obstetric outcomes. Int J Gynaecol Obstet.

[9] Hutcheon JA, Riddell CA, Strumpf EC, Lee L, Harper S (2017). Safety of labour and delivery following closures of obstetric services in small community hospitals. CMAJ.

[10] Darling EK, Lawford KMO, Wilson K, Kryzanauskas M, Bourgeault IL (2019). Distance from home birth to emergency obstetric services and neonatal outcomes: a cohort study. J Midwifery Womens Health.

[11] Ravelli AC, Jager KJ, de Groot MH, Erwich JJ, Rijninks-van Driel GC, Tromp M (2011). Travel time from home to hospital and adverse perinatal outcomes in women at term in the Netherlands. BJOG.

[12] Engjom HM, Morken NH, Hoydahl E, Norheim OF, Klungsoyr K (2017). Increased risk of peripartum perinatal mortality in unplanned births outside an institution: a retrospective population-based study. Am J Obstet Gynecol.

[13] Tracy SK, Sullivan E, Dahlen H, Black D, Wang YA, Tracy MB (2006). Does size matter? A population-based study of birth in lower volume maternity hospitals for low risk women. BJOG.

[14] Bernitz S, Rolland R, Blix E, Jacobsen M, Sjoborg K, Oian P (2011). Is the operative delivery rate in low-risk women dependent on the level of birth care? A randomised controlled trial. BJOG.

[15] Coulm B, Le Ray C, Lelong N, Drewniak N, Zeitlin J, Blondel B (2012). Obstetric interventions for low-risk pregnant women in France: do maternity unit characteristics make a difference?. Birth (Berkeley Calif).

[16] Schroeder L, Patel N, Keeler M, Rocca-Ihenacho L, Macfarlane AJ (2017). The economic costs of intrapartum care in Tower Hamlets: a comparison between the cost of birth in a freestanding midwifery unit and hospital for women at low risk of obstetric complications. Midwifery.

[17] Schroeder E, Petrou S, Patel N, Hollowell J, Puddicombe D, Redshaw M (2012). Cost effectiveness of alternative planned places of birth in woman at low risk of complications: evidence from the Birthplace in England national prospective cohort study. BMJ.

[18] Hinton L, Dumelow C, Rowe R, Hollowell J (2018). Birthplace choices: what are the information needs of women when choosing where to give birth in England? A qualitative study using online and face to face focus groups. BMC Pregnancy Childbirth.

[19] Grigg C, Tracy SK, Daellenbach R, Kensington M, Schmied V (2014). An exploration of influences on women’s birthplace decision-making in New Zealand: a mixed methods prospective cohort within the Evaluating Maternity Units study. BMC Pregnancy Childbirth.

[20] Hoang H, Le Q, Ogden K (2014). Women’s maternity care needs and related service models in rural areas: a comprehensive systematic review of qualitative evidence. Women Birth.

[21] Malouf RS, Tomlinson C, Henderson J, Opondo C, Brocklehurst P, Alderdice F (2020). Impact of obstetric unit closures, travel time and distance to obstetric services on maternal and neonatal outcomes in high-income countries: a systematic review. BMJ Open.

[22] Blok H, Broeders L, van Dijk AE, Koole S, Rosman AN, Vis LC. Perinatale zorg in Nederland anno 2019. Landelijke perinatale cijfers en duiding. Utrecht: Perined; 2020. https://assets.perined.nl/docs/aeb10614-08b4-4a1c-9045-8af8a2df5c16.pdf. Accessed 23 Jul 2021.

[23] Expert group Standard Integral Birth Care. Zorgstandaard Integrale Geboortezorg. Utrecht: Netherlands College of Perinatal Care; 2020. https://www.kennisnetgeboortezorg.nl/wp-content/uploads/2020/11/zorgstandaard-integrale-geboortezorg-1.2.pdf. Accessed 23 Jul 2021.

[24] Health and Youth Care Inspectorate. Veerkracht en betrokkenheid. Goede zorg na overdracht uit MC IJsselmeerziekenhuizen. Utrecht: Health and Youth Care Inspectorate; 2019. https://www.rijksoverheid.nl/documenten/rapporten/2020/01/07/veerkracht-en-betrokkenheid-goede-zorg-na-overdracht-uit-mc-ijsselmeerziekenhuizen. Accessed 23 Jul 2021.

[25] IG&H consultancy. Toekomstverkenning zorg in Flevoland. Utrecht: IG&H Consultancy; 2019. https://www.rijksoverheid.nl/documenten/rapporten/2019/07/12/toekomstverkenning-zorg-in-flevoland. Accessed 23 Jul 2021.

[26] Braun V, Clarke V (2006). Using thematic analysis in psychology. Qual Res Psychol.

[27] Knoster T, Villa R, Thousand J. A framework for thinking about systems change. Restruct Caring Eff Educ Piecing Puzzle Together. 2nd ed. Baltimore: Paul H. Brookes Publishing Co.; 2000. p. 93–128.

[28] Burnes B (2020). The origins of Lewin’s three-step model of change. J Appl Behav Sci.

[29] Brent MR. An attributional analysis of Kübler-Ross’ model of dying Cambridge. Cambridge: Harvard University; 1981.

[30] Tong A, Sainsbury P, Craig J (2007). Consolidated criteria for reporting qualitative research (COREQ): a 32-item checklist for interviews and focus groups. Int J Qual Health Care.

[31] Todnem By R (2005). Organisational change management: a critical review. J Change Manage.

[32] Cleary M, West S, Arthur D, Kornhaber R. Change management in health care and mental health nursing. Issues Ment Health Nurs. 2019;40(11):966-72. 10.1080/01612840.2019.1609633 31219727

[33] Nilsen P, Schildmeijer K, Ericsson C, Seing I, Birken S (2019). Implementation of change in health care in Sweden: a qualitative study of professionals’ change responses. Implementation Sci.

[34] Kremer JA, Ouwehand A, Hoff J. Van deelbelangen naar gedeeld belang. Den Haag: Dutch Council for Public Health and Society; 2020. https://www.raadrvs.nl/documenten/publicaties/2020/06/17/van-deelbelangen-naar-gedeeld-belang. Accessed 23 Jul 2021.

[35] Dijsselbloem JR, van Asselt MB, Zouridis S, Faillissement MC, Slotervaart en MC. IJsselmeerziekenhuizen. Risico’s voor patiëntveiligheid. Den Haag: Dutch Research Council for Safety; 2019. https://www.tweedekamer.nl/kamerstukken/detail?id=2019Z25976&did=2019D53366. Accessed 23 Jul 2021.

[36] Quality, Complaints and Disputes Act of the Netherlands, Article 11.1a. Den Haag: Official Gazette of the Kingdom of the Netherlands; 2015. https://zoek.officielebekendmakingen.nl/stb-2015-407.html. Accessed 23 July 2021.

[37] Bamberger SG, Vinding AL, Larsen A, Nielsen P, Fonager K, Nielsen RN (2012). Impact of organisational change on mental health: a systematic review. Occup Environ Med.

[38] Nanda A, Wasan A, Sussman J (2017). Provider health and wellness. J Allergy Clin Immunol Pract.

[39] Heaman MI, Sword W, Elliott L, Moffatt M, Helewa ME, Morris H (2015). Barriers and facilitators related to use of prenatal care by inner-city women: perceptions of health care providers. BMC Pregnancy Childbirth.

[40] Bailit J (2012). Impact of non-clinical factors on primary cesarean deliveries. Semin Perinatol.

[41] Perdok H, Jans S, Verhoeven C, van Dillen J, Batenburg R, Mol BW (2016). Opinions of professionals about integrating midwife- and obstetrician-led care in The Netherlands. Midwifery.

[42] De Jonge A, Downe S, Page L, Devane D, Lindgren H, Klinkert J (2019). Value based maternal and newborn care requires alignment of adequate resources with high value activities. BMC Pregnancy Childbirth.

[43] Sandall J, Soltani H, Gates S, Shennan A, Devane D (2016). Midwife-led continuity models versus other models of care for childbearing women. Cochrane Database Syst Rev.

