# Supplementary material for: Centralisation of acute obstetric care in the Netherlands: a qualitative study to explore the experiences of stakeholders with adaptations in organisation of care
Source: BMC Health Serv Res. 2021 Nov 13;21:1233. doi: 10.1186/s12913-021-07269-4 (PMC8590329; doi:10.1186/s12913-021-07269-4)
Supplement: Supplementary file 1 — Additional file 1. [file 12913_2021_7269_MOESM1_ESM.pdf]

**Title**	Centralisation of acute obstetric care in the Netherlands: a qualitative study to explore the experiences of maternity care stakeholders
**Date**	12/05/2020
**Researcher**	Lauri van den Berg<br>Master student Health Sciences
**Coordinating Researcher**	Prof. Ank de Jonge<br>Professor in Midwifery Science, location VUmc<br>Van der Boechorststraat 7<br>1081 BT Amsterdam
**Principal investigator**	Prof. Ank de Jonge<br>Professor in Midwifery Science, location VUmc<br>Van der Boechorststraat 7<br>1081 BT Amsterdam
**Department**	Amsterdam Public Health

**Rationale**	In recent years, centralisation of acute obstetric care (AOC) took place in several regions in the Netherlands. In response to this centralisation of AOC, stakeholders in maternity care have made adaptations in the organisation of care. These adaptations are still largely unknown. The aim of this qualitative study is to identify these adaptations and in particular the experiences of stakeholders with centralisation of AOC and the adaptations after that. Some adaptations might serve as an example for other regions with future centralisation plans.
**Goal**	Primary goal: knowing the experiences of maternity care stakeholders with centralisation of AOC and their adaptations in organisation of maternity care.<br>Secondary goal: this research might help regions with centralisation plans of AOC in the future.
**Study design**	Qualitative study with semi-structured in-depth interviews.
**Study population**	About fifteen stakeholders within the maternity care sector. They are all over the age of 18. The following stakeholders will definitely be approached: (clinical) midwives, obstetricians, maternity care assistant organisation, insurer, CPZ, inspectors of the Health and Youth Care Inspectorate and pregnant women or women who have recently given birth.
**Inclusion criteria**	• Working in or experience with one of the three selected regions: Drenthe, Flevoland and Nieuwegein.<br>• Stakeholder within the maternity care sector.<br>• Patient must have recent experience with maternity care after centralisation of AOC.
**Exclusion criteria**	• Age below 18
**Sample size**	Fifteen. This number was chosen because of the limited available time and because it is expected that by that time data saturation has been reached.
**Recruitment participants**	The participants are approached via e-mail to participate in the interview. They receive an information letter. If they wish to participate, the study and their rights are explained before the

	interview starts. They then sign the informed consent statement.
**Intervention**	1 hour in-depth interview
**Standard treatment**	NA
**Study parameters**	It is a qualitative study and because we use inductive analysis, a primary outcome measure is not applicable.
**Statistical analysis**	NA<br>Qualitative content analysis in Atlas.ti.
**Investment for the research participant**	1 hour time investment
**Risk for the research participant**	No risk for the participant.
**Benefits of involvement for the research participant**	A conversation about the topic. Possibly new insights on the subject.
**Disadvantages for the research participant**	1 hour time investment
**Compensation for the research participant**	No compensation
**Administrative organisation**	The interviews are recorded and transcribed by the researcher. After transcription, the recordings are deleted. The transcripts are stored on a secure server and are not accessible to anyone other than the researchers.
