# Supplementary material for: Centralisation of acute obstetric care in the Netherlands: a qualitative study to explore the experiences of stakeholders with adaptations in organisation of care
Source: BMC Health Serv Res. 2021 Nov 13;21:1233. doi: 10.1186/s12913-021-07269-4 (PMC8590329; doi:10.1186/s12913-021-07269-4)
Supplement: Supplementary file 2 — Additional file 2. [file 12913_2021_7269_MOESM2_ESM.pdf]

Care providers and other stakeholders (patients excluded)			
**Topics**	**Sub topics**	**Main question(s)**	**Possible follow-up questions**
Introduction<br>10 min.	• Degree of involvement in the centralisation of acute obstetric care<br>• Venting emotions	Can you tell us more about yourself?	• How many years of work experience do you have?<br>• To what extent are you affected by the closure of the acute obstetric care unit in your organisation? Or as a health care provider? And as a person?<br>• Were you part of any committees/working groups or did you attend any meeting that dealt with the closure of an acute obstetric care unit?<br>○ If so, which one?<br>○ If not, how were you informed about updates?<br>• Can you tell something about what this subject (centralisation of acute obstetric care) brings up for you? (emotions)
Centralisation of acute obstetric care	• Experiences opportunities<br>• Perceived risks	• What opportunities did you see when the closure of the acute	• One of the recommendations of the report 'Een goed begin' was 24/7 acute obstetrics. Centralisation of acute

<u>10 min.</u>		obstetric care ward was announced?<br>• What potential risks were you most concerned about after the announcement of the closure of the acute obstetric care unit?	obstetric care could be a way to achieve this. What is you vies on this?
Adaptations in the organisation of care after centralisations<br><u>25 min.</u>	• Macro/meso/micro level<br>• Related to perceived risks<br>• Alternatives<br>• Usability for other regions	• What adaptations in the organisation of maternity care did you make as …/ do you see in your role as … after an acute obstetric care unit was closed?	• Can you tell me how the process of making adaptations went?<br>• What do you consider important when there are changes in the organisation of maternity care?<br>• What is your experience with the adaptations you mentioned?<br>• In your opinion, are the adaptations you mentioned temporary or future-proof and why?<br>• Are the adaptations you mentioned specific to your/a particular region?<br>○ Within the MCC, there are regional differences, such as a different population and

			different perinatal outcomes. Are you aware of this? And does this require different adaptations per municipality?<br>• Could the adaptations you mentioned work in other regions as well? Why or why not?<br>• Would/Which other adaptations would have been possible in your opinion?<br>• What has turned out to be important (in retrospect) in the process of making adaptations?<br>• What tips/tricks would you give other organisations/health care providers when they are confronted with centralisation of acute obstetric care plans?<br>• A possible adaptation was also this: … Would this have been applicable to your region and why do you think this is a good adaptation or not?

			• Ask for another level of adaptations (macro/meso/micro). E.g. The adaptations you mention are mainly within your organisation. To what extent have you cooperated with other organisations (e.g. health care insurance, municipality, MCC) to make adaptations?<br>• [Maternity care providers] You and your organisation are members of the MCC. At the moment, the MCC is often not a formal discussion partner in consultations on proposed centralisation of acute obstetric care. How was your experience regarding this?<br>○ Did you experiences support from the MCC when there where centralisation plans?<br>• [Health insurance company]<br>○ Would you advocate for regional or national adaptations

			in organisation of maternity care after centralisation of acute obstetric care?<br>○ How do you consider the role of the health care insurance in helping with adaptations in organisation of care. Such as:<br>▪ Financing extra ambulances<br>▪ Eliminating extra fee for hospital birth without medical indication<br>▪ Financing transportation costs for insured persons with financial problems<br>▪ H3 regulation<br>○ How is the cooperation with other health care insurances when it comes to making adaptations in the organisation of maternity care?

			• [CPZ]<br>○ How do you describe your role as CPZ when there are centralisation plans?<br>○ “Do you have a place at the discussion tables?”<br>○ Why do you think this is or isn’t your job?
Sharing ‘good practices’<br><u>5 min.</u>	• Useful?<br>• By whom?	To what extent would the sharing of good examples be useful for regions with centralisation plans?	○ Could the IGJ play a role in sharing good practices when it comes to adaptations in the organisation of maternity care after centralisation?<br>▪ Or another organisation such as CPZ or ROAZ?
Closure of the interview<br><u>10 min.</u>	• Member check	This is, summarised, what I understood from you during the interview: ….	• Do you have anything to add or clarify as a result of this summary, for example, new thoughts or ideas that you would like to discuss?

Patients			
**Topics**	**Sub-topics**	**Main question(s)**	**Possible follow-up questions**
Introduction<br>10 min.	• Degree of involvement in the subject<br>• Personal experiences	• Can you tell something more about yourself/Can you introduce yourself?	• What us your personal experience of the closure of the acute obstetric care department in your region?
Quality of care<br>5 min.		• To what extent are you willing to travel longer to a hospital if the results or facilities (e.g. NICU) are better there? For example: better care for a baby with a difficult start?	• How much longer are you willing to travel for better care?<br>• What does it depend on whether you are prepared to travel longer?
Changes in the organisation of maternity care<br>15 min.		• What is important to you when changes are made to the organisation of maternity care?	• In your opinion, what has changed in the care you received since closure of the acute obstetric care department?<br>○ What is your opinion on these changes?<br>○ How were you informed about changes in the organisation of maternity care?

			• What do you think should change in the organisation of maternity care after delivery rooms are closed?<br>○ Why do you think so?<br>○ What role do patients have in your proposed changes?
adaptations made by patients themselves<br><u>20 min.</u>	• Own adaptations, and why?<br>• Experiences in the surroundings of the pregnant woman or woman who has recently given birth?	• Have you made any adaptations of changed to your choices about your pregnancy, birth and post-partum period because the acute obstetric care unit in your regions closed?<br>• What adaptations or choices?	• Are you uncomfortable with the closure of the acute obstetric care department in your region?<br>• Does travel distance plays a role in your choice to give birth at home or not?<br>○ If the travel distance increases are you more likely to opt for a home birth or a hospital birth?<br>○ How important is it for you to have the choice between home or hospital birth?<br>• What do you hear from other pregnant women or new moms in your area? What are their experiences, concerns, opinions and/or adaptations?

Closure of the interview<br><u>10 min.</u>	• Member check	This is, summarised, what I understood from you during the interview: ….	• Do you have anything to add or clarify as a result of this summary, for example, new thoughts or ideas that you would like to discuss?
